# Supplementary material for: Immortal genome assumption significantly underestimates replication and death rates of Mycobacterium tuberculosis in mice and monkeys
Source: Microbiol Spectr. 2026 Jun 15;14(7):e03020-25. doi: 10.1128/spectrum.03020-25 (PMC13340352; doi:10.1128/spectrum.03020-25)
Supplement: Supplemental material — Additional mathematical considerations, decay of Mtb in soil, and Fig. S1 to S8. [file spectrum.03020-25-s0001.pdf]

Immortal genome assumption significantly underestimates replication and death rates of *Mycobacterium tuberculosis* in mice and monkeys

Allan D. Friesen and Vitaly V. Ganusov

Supplemental Information

S1 Additional mathematical considerations

S1.1 General Model

We developed a general model (**eqns. (3)–(5)**) of dynamics of CFUs and CEQs that includes the following components and processes (see **Figure 1**A and **eqns. (3)–(5)**):

- Subpopulations B and D represent culturable bacteria and unculturable bacteria, respectively. Unculturable subpopulation D includes both VBNC bacteria and chromosomes of dead bacteria.
- Culturable bacteria replicate with average per capita rate r.
- Culturable bacteria leave the culturable state with per capita rate $\delta_B + \delta_{B^*}$, where δ_B is the conversion rate of B to dead and/or dormant state D, with (temporary) preservation of the genome, and δ_{B^*} is the rate of bacteria death with simultaneous degradation of the genome (for example, by phagocytosis).
- Bacteria in state D may replicate with per capita rate r_D, which cannot exceed r_B.
- Genomes in subpopulation D are degraded with per capita rate δ_D.

The dynamics of CFUs/CEQs = Z in the general model follow a logistical form:

$$\frac{\mathrm{d}Z}{\mathrm{d}t} = \rho Z \left(1 - \frac{Z}{Z_\infty}\right), \tag{S.1}$$

where

$$\rho = r - \delta_B - \delta_{B^*} - r_D + \delta_D, \tag{S.2}$$

$$Z_\infty = \frac{\rho}{\rho + \delta} = \frac{r - \delta_B - \delta_{B^*} - r_D + \delta_D}{r - \delta_{B^*} - r_D + \delta_D}. \tag{S.3}$$

S1.2 Independent Dynamics model

S1.2.1 Interpretation of ID model parameters

Intermediate between the general model and the independent dynamics (**ID**) models we can define the flexible independent dynamics model (**FID**, **eqns. (11)–(12)**), obtained from the general model by setting $\delta_Q = \delta_{B^*} = \delta_D$ (**eqns. (12)–(11)**). In this model, the populations B and Q each have their own replication and death rates, resulting in 6 adjustable parameters including initial conditions. In order to fit experimental data unambiguously, an additional constraint is needed. The ID model is a special case of the FID model with the restriction that populations B and Q share the same replication rate, $r = r_B = r_Q$. During early infection, this is a natural

choice, since we expect $B = Q$ at that point. It is possible that some other choices may make better sense later during infection. For example, the analysis of Lin *et al.*[13] appears to suggest that $r_Q = r \approx 0$ after about 4 weeks of infection, although this is not entirely clear, since they do not list parameters.

In the ID model, r clearly represents the replication rate of viable bacteria; however, requiring that this constant also defines the replication rate of Q, the ID model in its restricted form implies that subpopulation Q is composed of a combination of platable and VBNC bacteria, all replicating with rate r. The rate δ cannot be thought of as representing a death rate in this model, since the underlying mechanism implies that the non-platable bacteria can replicate.

This difficulty is present even in the more flexible FID model. To see this, parameters of the FID and the DD models can be related to each other. The rate r_Q is simply the weighted average of r_B and r_D:

$$r_Q = \frac{rB + r_D D}{B + D} = \frac{rB + r_D(Q - B)}{Q} = r_D + (r - r_D)\frac{B}{Q}, \tag{S.4}$$

and δ_Q is conceptually similar to δ_D, except that it represents an average decay rate of *all* genomes, rather than just those of dead bacteria:

$$\delta_Q = \delta_D \frac{D}{B + D} = \delta_D \left(1 - \frac{B}{Q}\right). \tag{S.5}$$

Because the difference $Q - B$ changes over time, the parameters of both models cannot generally be taken simultaneously to be time-independent. The result of this analysis is that independent fitting of exponential (or logistical) functions to B and Q produces parameters that cannot be associated in a simple way with replication or death rates of particular populations.

S1.3 The Lin et al. model is not consistent with division of CEQs into platable and dead populations

The model of Lin *et al.*[13] (**eqns.** (1)–(2)) applies separate logistical forms for dynamics of CFUs and CEQs of Mtb in lesions from the lungs of macaques. Early during infection, the CFUs and CEQs are far smaller than their corresponding carrying capacities, so that the dynamics of this model is equivalent to that of the ID model. As such, it is also subject to the same difficulties with associating the rates with replication and death, as well as with the assumption that the VBNC population is negligibly small. However, if replication and death rates are allowed to change arbitrarily over time, one can derive their time dependence under the assumption that the CEQs divide cleanly into dead and platable bacteria, by application of **eqns.** (**S.24**)–(**S.25**) to the curves (**eqns.** (1)–(2)). The result is that both replication and death rates increase exponentially as B begins to saturate, then both rates decrease exponentially as Q saturates.

S1.3.1 Dynamics of CFUs/CEQs in FID and ID models

For the FID model, the dynamics of Z = CFUs/CEQs follow exponential decay at a rate that depends on replication and death rates of CFUs and CEQs:

$$\frac{\mathrm{d}Z}{\mathrm{d}t} = -\left[(\delta - \delta_Q) - (r_B - r_Q)\right] Z. \tag{S.6}$$

Because the rate of exponential decay depends on all four rates, association of the dynamics of Z with particular biological processes is difficult, without added assumptions. The ID model's simplifying assumption that $r_B = r_Q$ leads to the simpler result,

$$\frac{\mathrm{d}Z}{\mathrm{d}t} = -(\delta - \delta_Q)Z. \tag{S.7}$$

Under this assumption, decay of CFUs/CEQs reflects the difference between δ and the genome decay rate δ_Q. If additionally, δ_Q were negligibly small, the dynamics of Z could be associated with the death/decay rate δ of CFUs.

S1.4 Dependent Dynamics model

S1.4.1 Interpretation of DD model parameters

Starting again from the general model, setting $\delta_{B^*} = 0$ and $r_D = 0$ leads to the DD model, defined by **eqns.** (8)–(10). Because the DD model contains explicit live and dead subpopulations in its formulation, interpretation of parameters in the DD model is simple: r represents the per capita replication rate of viable bacteria, δ represents the per capita death rate, and δ_D represents the per capita decay rate of detectable genomes of dead bacteria. It is also straightforward to allow some of the population D to replicate by introducing a small replication rate, r_D to the model. Additional data would be needed to justify addition of this parameter. r_D would, in principle, represent the average per capita replication rate of bacteria in subpopulation D; similarly, δ_D would now represent a degradation rate of genomes, averaged over both VBNC and dead populations. In such a case, the rates r_D and δ_D would be expected to vary over time, even when r and δ are constant, since the proportion of D that represents dead bacteria would most likely change over time. A model that explicitly distinguishes VBNC and dead populations would seem more realistic, though such a model likely would require additional fitting parameters, increasing the chance of overfitting.

S1.4.2 Dynamics of CFUs/CEQs in DD model

Similar to the general model, the dynamics of CFUs/CEQs = Z in the DD model follow a logistical equation:

$$\frac{\mathrm{d}Z}{\mathrm{d}t} = (r - \delta + \delta_D)Z - (r + \delta_D)Z^2 = \rho Z\left(1 - \frac{Z}{Z_\infty}\right), \tag{S.8}$$

where net rate ρ and carrying capacity Z_∞ are given by:

$$\rho = r - \delta + \delta_D, \tag{S.9}$$

$$Z_\infty = \frac{r - \delta + \delta_D}{r + \delta_D} = 1 - \frac{\delta}{r + \delta_D}. \tag{S.10}$$

When bacterial numbers are increasing, Z saturates at Z_∞. However, when bacterial numbers are decreasing, Z_∞ is typically negative, so that the trajectory of Z instead approaches an exponential decay (**Supplemental Figure S1**).

During chronic infections, the net rate ρ is small, so that the first order term in **eqn. (S.8)** is small, and the long-time limit of Z is close to zero. In this case, the dynamics of Z are approximately second order.

Integration of the DD model gives the following forms for the dynamics of CFUs, B, dead bacteria genomes, D, and their ratio, Z:

$$B = B_0 e^{(r-\delta)t}, \tag{S.11}$$

$$D = \frac{\delta B_0}{\rho}\left(e^{\rho t} - 1\right) e^{-\delta_D t} + D_0 e^{-\delta_D t}, \tag{S.12}$$

$$Z = \frac{Z_\infty}{1 + \left(\frac{Z_\infty - Z_0}{Z_0}\right) e^{-\rho t}},, \tag{S.13}$$

where ρ and Z_∞ are defined as above.

When the viable population declines faster than the decay rate δ_D of detectable genomes of killed bacteria, the carrying capacity, Z_∞, becomes negative. In this case, the long-time dynamics of Z asymptotically approach an exponential decay, described by the limiting behavior, Z_L, of **eqn. (S.13)** when t is large and ρ is negative (**Supplemental Figure S1**):

$$Z_L = \frac{Z_\infty Z_0}{Z_\infty - Z_0} e^{\rho t}, \tag{S.14}$$

where as before, $\rho = r - \delta + \delta_D$. When considered over a sufficiently long time, the full dynamics of decline of Z in the DD model will be approximated well by **eqn. (S.14)**. In this case, the difference in per capita rates of decline of Z and B provide an estimate of δ_D:

$$\frac{\mathrm{d}Z}{\mathrm{d}t}/Z - \frac{\mathrm{d}B}{\mathrm{d}t}/B \approx (r - \delta + \delta_D) - (r - \delta) = \delta_D. \tag{S.15}$$

Use of this approximation slightly biases toward underestimate of δ_D, so that it may be thought of as a method for estimating a *lower bound* on the degradation rate of Mtb genomes in vivo. However, this method only works if the dynamics of CFUs and CFU/CEQ ratio approaches an asymptote, e.g., for constant (time-independent) parameters; if the rate of Mtb replication and death change over time, this approximation may give incorrect estimate of the CEQ genome rate. As an example, we estimated the change in $\log Z = -0.104$/day and $\log B = -0.056$/day between 4 and 11 weeks in Mtb-infected macaques[13]; this gave a negative estimate of the genome decay rate $\delta_D = -0.104 - (-0.056) = -0.048$/day highlighting the issue.

S1.5 Expressions for Estimating Model Parameters from Experimental Data

Ideally, rates would be extracted from time series including several different points. However, since studies usually include only two or three time points, it is more realistic to consider how

parameters can be estimated from just a pair of points. In these analyses we assume that the CEQ decay rate is known.

For the ID model, r and δ can be estimated from the following expressions:

$$r = \frac{\Delta \ln Q}{\Delta t} + \delta_Q, \quad (S.16)$$

$$\delta = r - \frac{\Delta \ln B}{\Delta t}. \quad (S.17)$$

Likewise, for the DD model,

$$r = (r - \delta + \delta_D)\frac{Q - Q_0 e^{-\delta_D t}}{B - B_0 e^{-\delta_D t}} - \delta_D = \rho \frac{\Delta Q_{\text{eff}}}{\Delta B_{\text{eff}}} - \delta_D, \quad (S.18)$$

$$\delta = (r - \delta + \delta_D)\left(\frac{Q - Q_0 e^{-\delta_D t}}{B - B_0 e^{-\delta_D t}} - 1\right) = \rho \left(\frac{\Delta Q_{\text{eff}}}{\Delta B_{\text{eff}}} - 1\right), \quad (S.19)$$

where

$$\Delta X_{\text{eff}} = X - X_0 e^{-\delta_D t}, \qquad X = B \text{ or } X = Q, \quad (S.20)$$

$$\rho = \frac{\Delta \ln B}{\Delta t} + \delta_D. \quad (S.21)$$

The following expressions could be used to produce the corresponding r and δ as a function of time for the for the ID model:

$$r = \frac{\frac{\mathrm{d}Q}{\mathrm{d}t}}{Q} + \delta_Q, \quad (S.22)$$

$$\delta = \frac{\frac{\mathrm{d}Q}{\mathrm{d}t}}{Q} - \frac{\frac{\mathrm{d}B}{\mathrm{d}t}}{B} + \delta_Q, \quad (S.23)$$

And for the DD model:

$$r = \frac{\frac{\mathrm{d}Q}{\mathrm{d}t}}{B} + \delta_D\left(\frac{Q}{B} - 1\right), \quad (S.24)$$

$$\delta = \frac{\frac{\mathrm{d}Q}{\mathrm{d}t} - \frac{\mathrm{d}B}{\mathrm{d}t}}{B} + \delta_D\left(\frac{Q}{B} - 1\right). \quad (S.25)$$

Note that while we have carried out this analysis for both ID and DD models, for completeness, the meaning of time-dependent r and δ in the ID model is unclear since r and δ are already aggregate variables, implying time-dependent replication and death rates.

S2 Decay of viable Mtb in soil

We digitized measurements of Mtb CFUs isolated from soil samples at 1 month intervals for 12 months after inoculation with a defined number of Mtb bacteria (Figure 1 of Ghodbane *et al.*[35]).

We found that the viable population declines in two phases. During the first two months after inoculation (phase 1), CFUs/g declined with a half-life of about 4.9 days (decay rate $d_1 = 0.14$/day). Subsequent decay in months 10-12 (phase 2) was substantially slower, with a half-life of about 36 days (decay rate $d_2 = 0.019$/day). Assuming a single exponential decay and fitting both slope and intercept resulted in an estimated half-life of 22 days (decay rate $d = 0.032$/day) that is quite similar to our estimated half-life time of CEQs in mice. If, instead, the intercept was fixed to the initial value of 10^8 CFUs/g, the resulting slope was steeper, corresponding to a half-life of about 14 days (decay rate $d = 0.050$/day).

S3 Additional figures

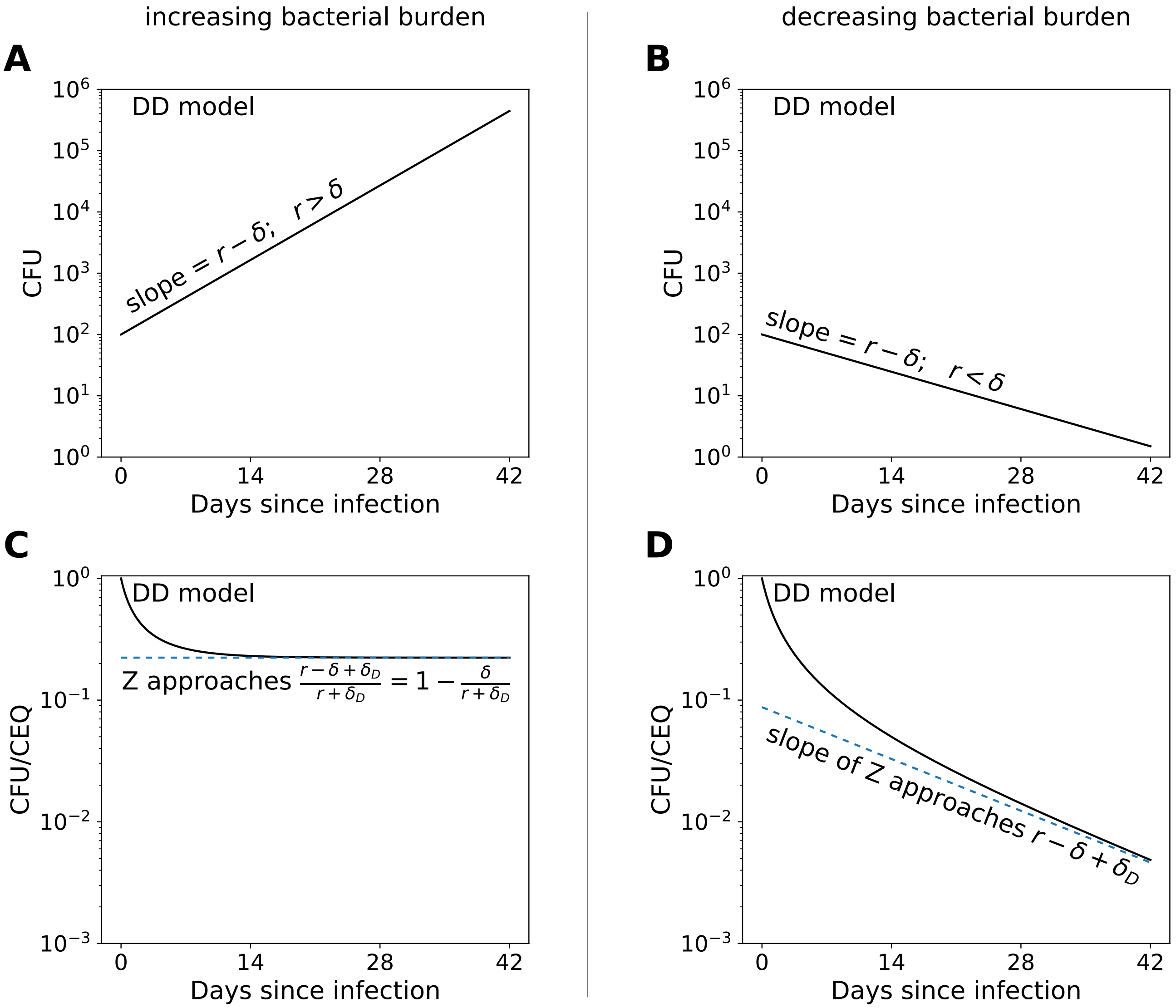

Supplemental Figure S1: DD model predicts different asymptotic behavior for CFU/CEQ ratio Z for expanding vs. contracting bacterial populations. We show predictions of the DD model (**eqns. (8)–(10)**) when bacterial population grows in size ($r > \delta$, panels **A** and **C**) or when it declines in size ($r < \delta$, **D** and **D**). More precisely, when $r + \delta_D > \delta$, Z approaches a positive carrying capacity (**C**). When $r + \delta_D < \delta$, the dynamics of Z approach the asymptotic limit, $Z_L = (1 - 1/Z_\infty))^{-1} \exp(\rho t)$ (**D**). In these simulations, parameters of the model are $r = 1$ day^{-1} (panels A and C), $r = 0.7$ day^{-1} (panels B and D), $\delta = 0.8$ day^{-1}, $\delta_D = 0.03$ day^{-1}, $B_0 = 10^3$, and $D_0 = 0$.

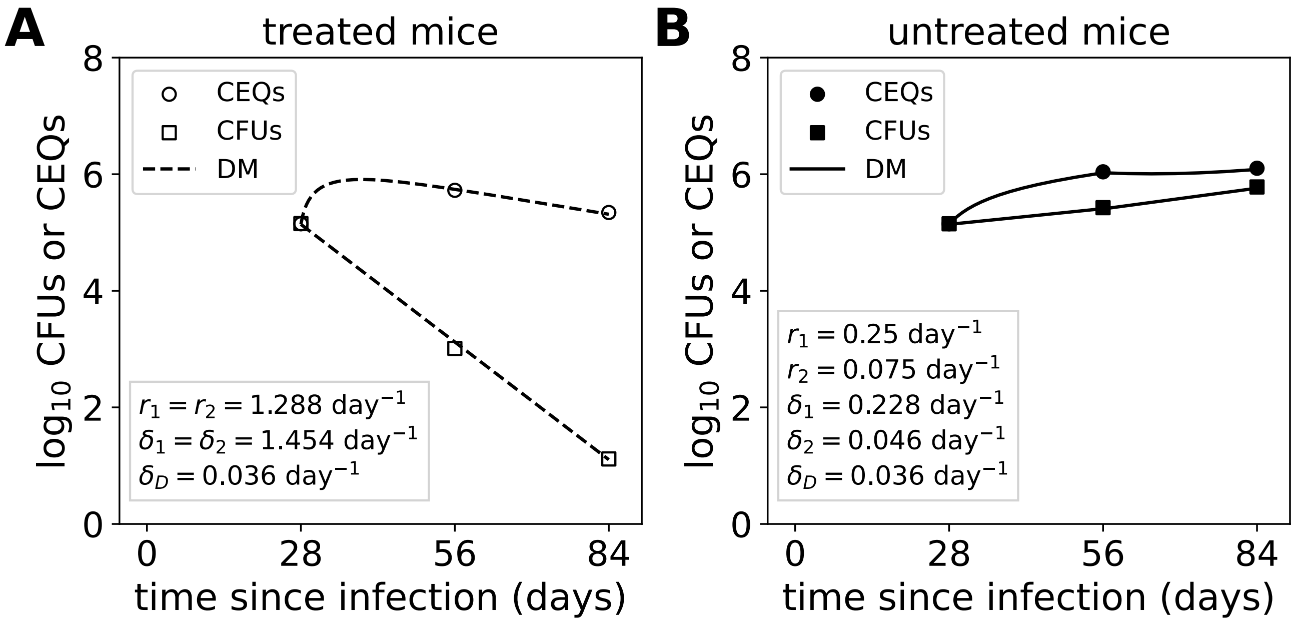

Supplemental Figure S2: Using DD model to estimate genome decay rate δ_D from CFU and CEQ data in mice. We fitted DD model (**eqns. (8)–(10)**) to CFU and CEQ lung data in INH-treated (**A**) or untreated (**B**) B6 mice infected with a high dose of Mtb intravenously[6]. In both panels, rates with subscript 1 or 2 represent the rates during the first (28-56 days) and second (56-84 days) time intervals, respectively; note that these intervals are different from those given in **eqn. (13)**. In panel **A**, only one set of rates was used, since best fits with two rates produced replication and death rates that differed by less than 2% between the two time intervals. Note that a large replication rate was required to fit the substantial rise in CEQs between 28 and 56 days post-infection, for treated mice. We initially fit the data with all parameters being free; we found that the best fit produced $\delta_D = 0.035$ day^{-1}. In panel **B**, we fixed the CEQ decay rate in the DD model to $\delta_D = 0.036$/day and estimated other model parameters are listed on the panel.

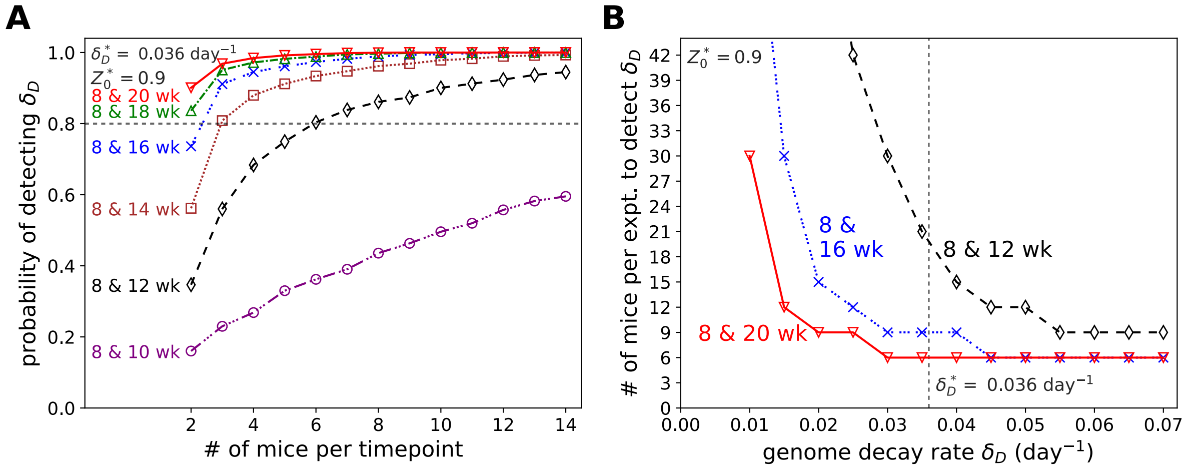

Supplemental Figure S3: Power analysis to detect Mtb genome decay rate δ_D. Similar analyses as in **Figure 4** except $Z(28) = 0.9$.

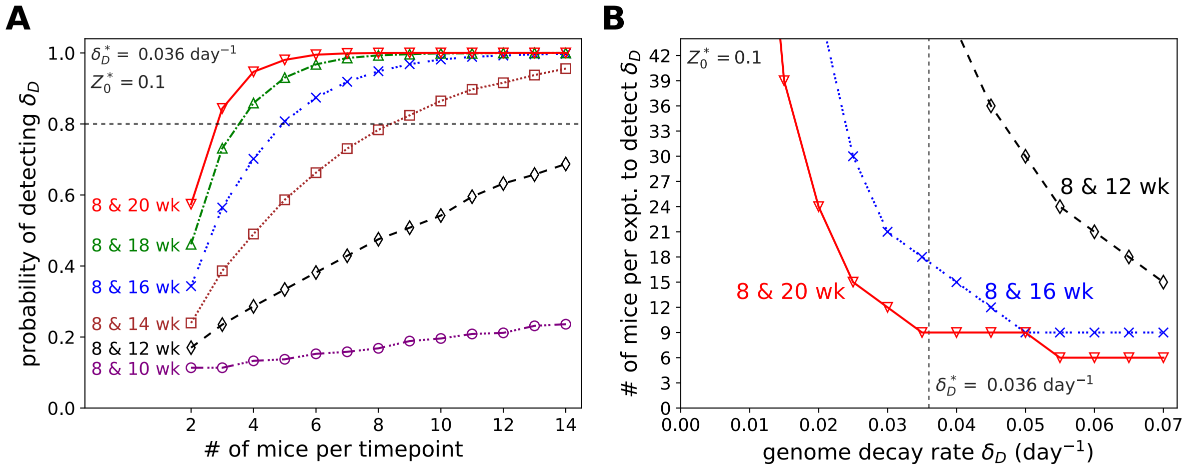

Supplemental Figure S4: Power analysis to detect Mtb genome decay rate δ_D. Similar analyses as in **Figure 4** except $Z(28) = 0.1$.

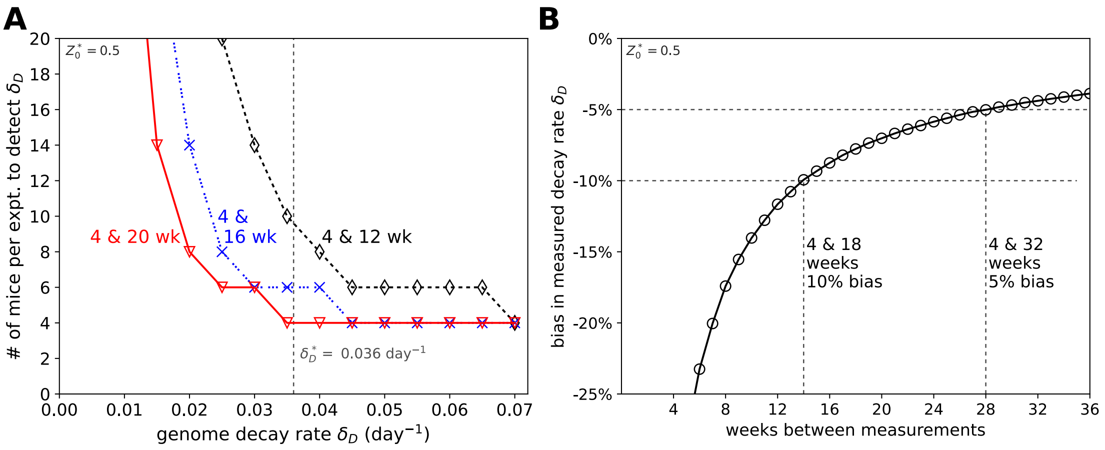

Supplemental Figure S5: An alternative experiment has higher statistical power but at the cost of introducing bias at estimating Mtb genome decay rate. In this alternative experiment, B and Q are measured only twice: the first is 28 days post-infection, before antibiotic treatment starts, and the second is at the end of the experiment; thus, this set-up requires $2n$ mice (see **Figure 4**A for comparison). **A**: Number of mice needed for 80% statistical power to detect genome decay rate of $\delta_D = 0.036$/day. **B**: Estimated percent bias predicted in measurement of δ_D. We estimated the bias by simulating the system with the DD model, with $\delta_D = 0.036$ day^{-1}. In simulations we assume that $Z(28) = 0.5$.

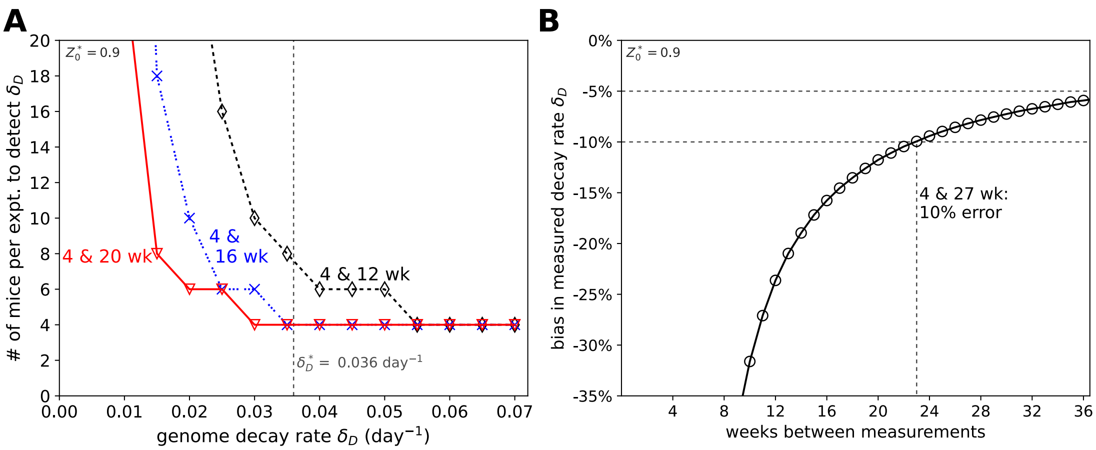

Supplemental Figure S6: An alternative experiment has higher statistical power but at the cost of introducing bias. Similar as in **Supplemental Figure S5** except we assume $Z(28) = 0.9$.

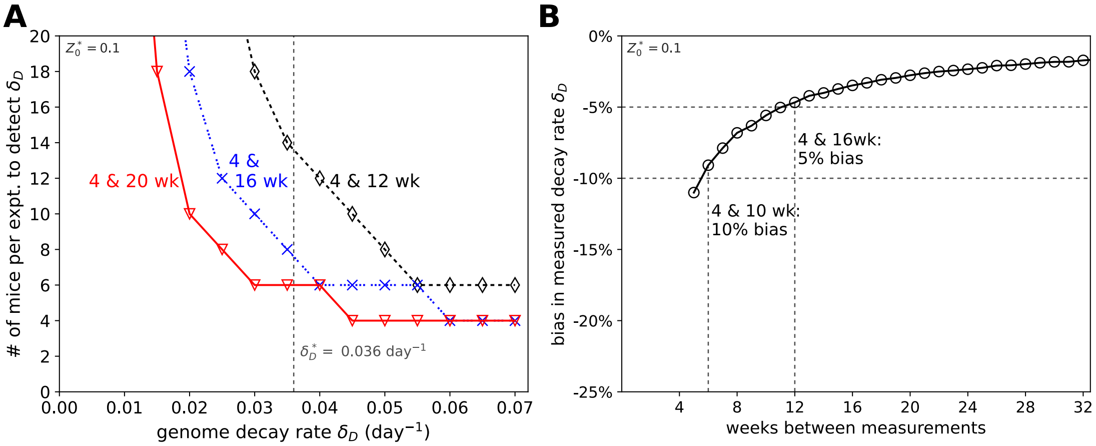

Supplemental Figure S7: An alternative experiment has higher statistical power but at the cost of introducing bias. Similar as in **Supplemental Figure S5** except we assume $Z(28) = 0.1$.

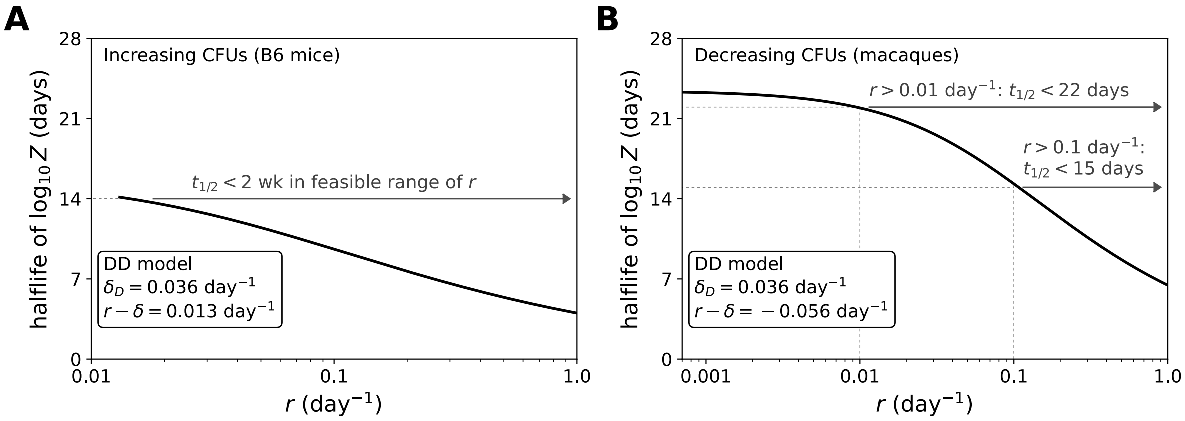

Supplemental Figure S8: Relatively rapid decay of detectable chromosomes results in a short memory of the CFU/CEQ ratio, when bacterial burdens are slowly increasing. We used the DD model to estimate the timescale over which dynamics of Z approach asymptotic behavior. **A**: To find the time for the difference $\log Z - \log Z_\infty$ to decay to half its initial value in mice between 4 and 8 weeks post-infection, we solved the equation $\log Z(t) - \log Z_\infty = \frac{1}{2}(\log Z(0) - \log Z_\infty)$ for t, where $Z(t)$ is given by **eqn. (S.13)**, and $Z_\infty = 1 - \delta/(r + \delta_D)$. We assumed that $Z(0) = 1$, and that $r - \delta = 0.013$ day^{-1}, the net growth rate we estimate in mice between 28 and 56 days post-infection (**Table 1**). **B**: To consider the timescale when the population is declining, we found the time for $\log Z - \log Z_L$ to decay to half its initial value, by iteratively solving $\log Z(t) - \log Z_L(t) = \frac{1}{2}(\log Z(0) - \log Z_L(0))$ for t, where Z_L is given by **eqn. (S.14)**. We again used $Z(0) = 1$, and we set $r - \delta = -0.055$ day^{-1}, the net rate of population decline Mtb in macaques, between 28 and 77 days post infection (**Table 2**).
